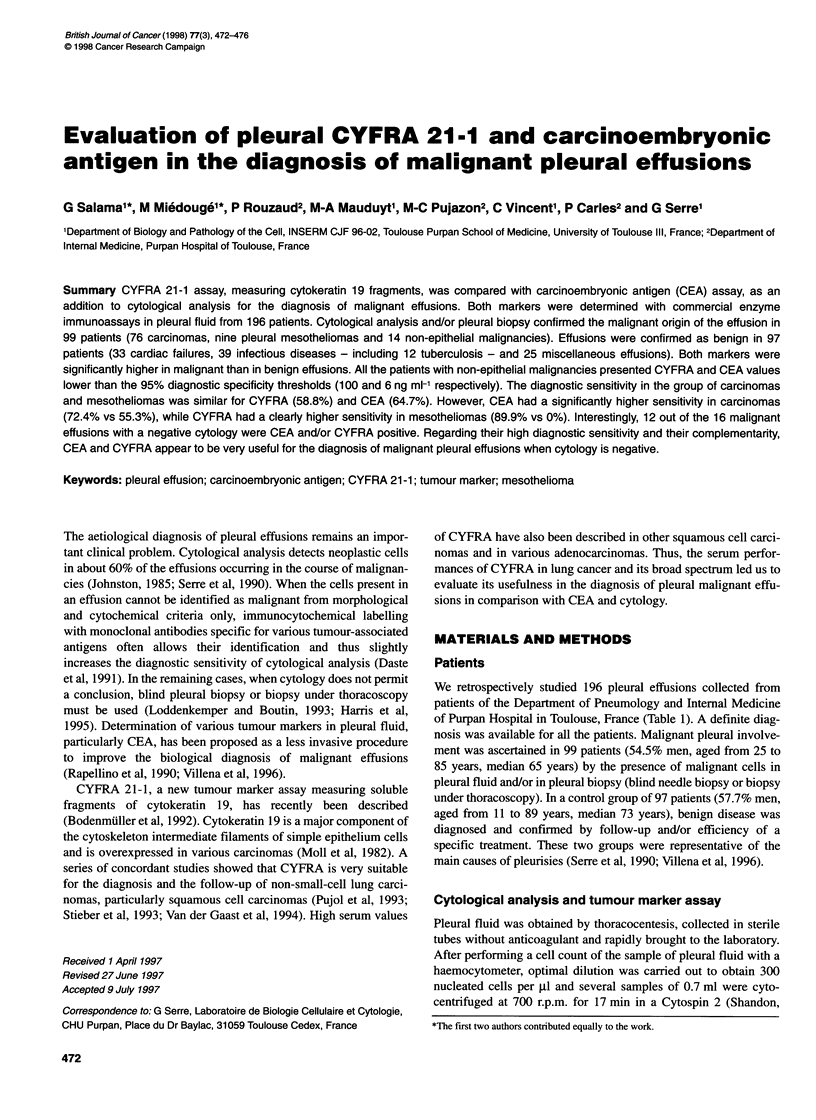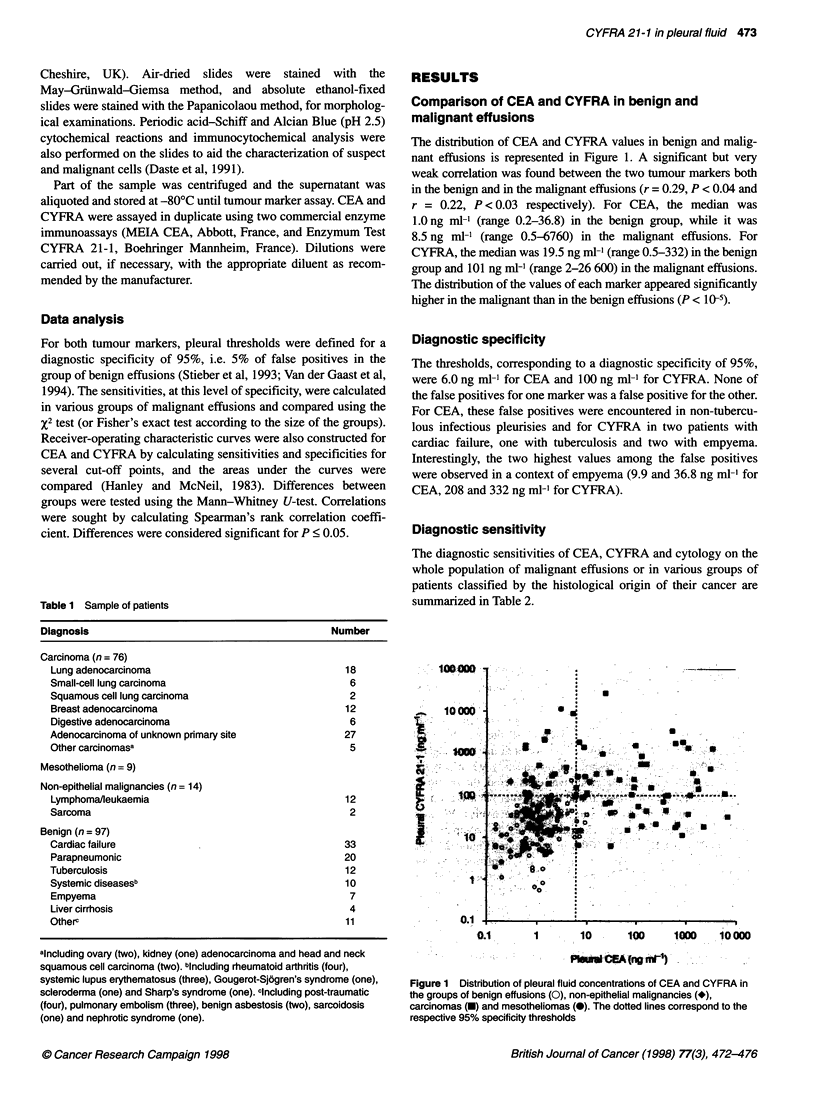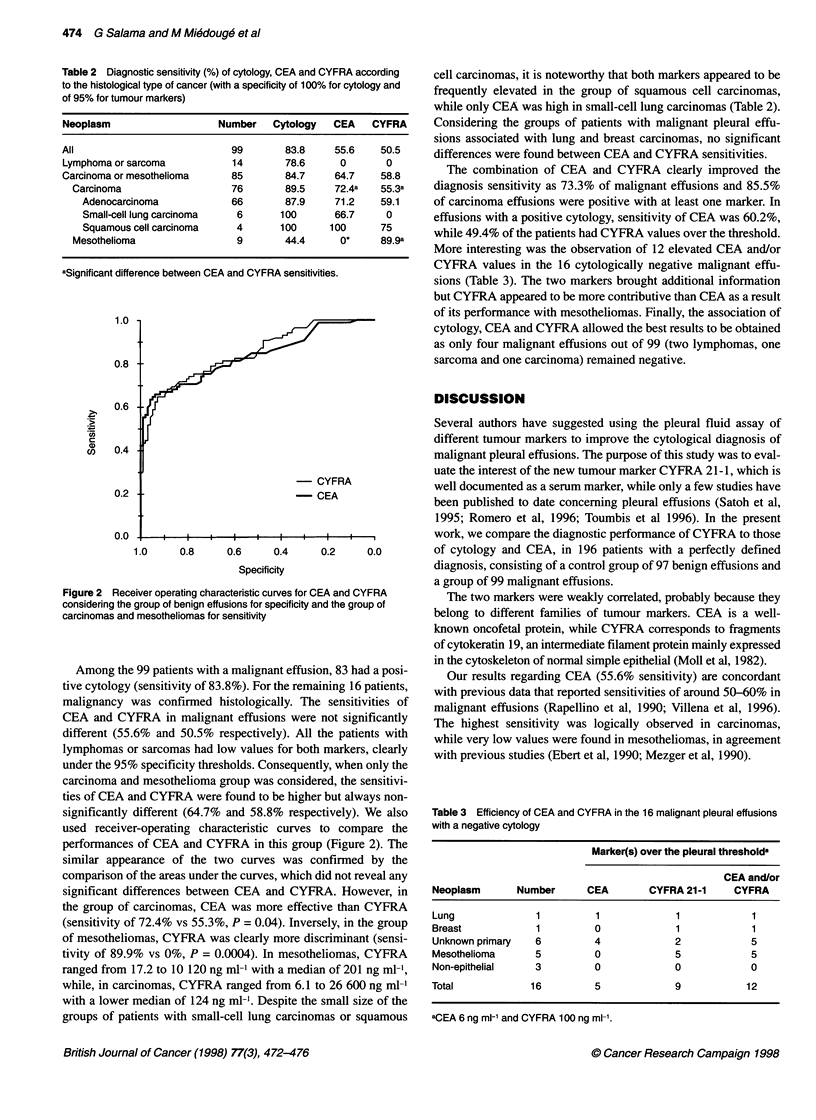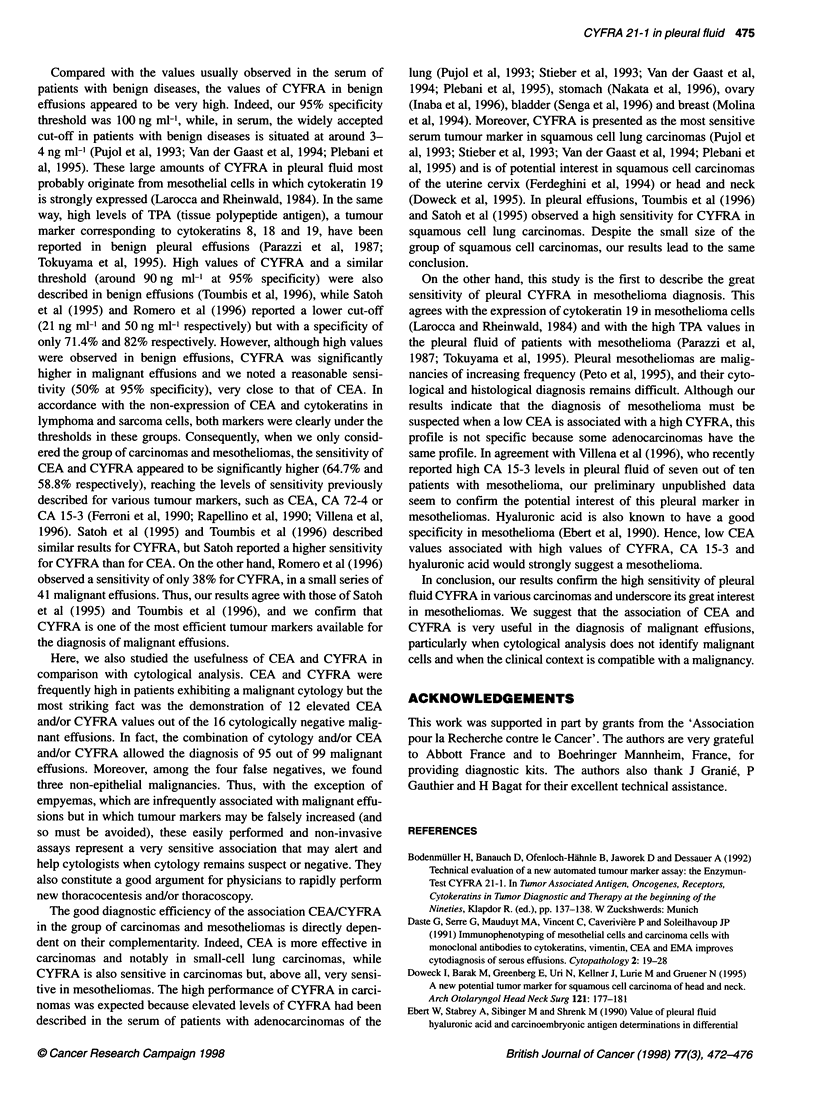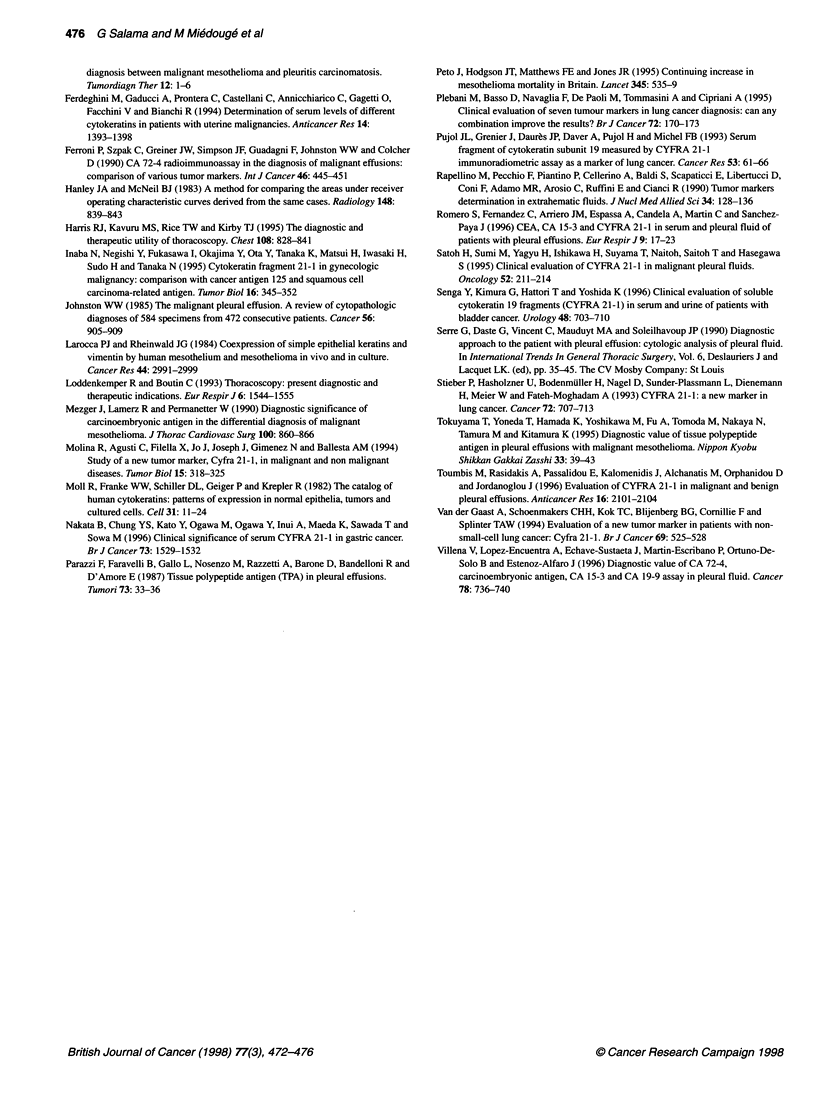# Evaluation of pleural CYFRA 21-1 and carcinoembryonic antigen in the diagnosis of malignant pleural effusions.

**DOI:** 10.1038/bjc.1998.75

**Published:** 1998

**Authors:** G. Salama, M. MiÃ©dougÃ©, P. Rouzaud, M. A. Mauduyt, M. C. Pujazon, C. Vincent, P. Carles, G. Serre

**Affiliations:** Department of Biology and Pathology of the Cell, INSERM CJF 96-02, Toulouse Purpan School of Medicine, University of Toulouse III, France.

## Abstract

**Images:**


British Journal of Cancer (1998) 77(3), 472–476
© 1998 Cancer Research Campaign

### Evaluation of pleural CYFRA 21-1 and carcinoembryonic antigen in the diagnosis of malignant pleural effusions

G Salama[1*], M Miédougé[1*], P Rouzaud[2], M-A Mauduyt[1], M-C Pujazon[2], C Vincent[1], P Carles[2] and G Serre[1]

[1]Department of Biology and Pathology of the Cell, INSERM CJF 96-02, Toulouse Purpan School of Medicine, University of Toulouse III, France; [2]Department of Internal Medicine, Purpan Hospital of Toulouse, France

**Summary** CYFRA 21-1 assay, measuring cytokeratin 19 fragments, was compared with carcinoembryonic antigen (CEA) assay, as an addition to cytological analysis for the diagnosis of malignant effusions. Both markers were determined with commercial enzyme immunoassays in pleural fluid from 196 patients. Cytological analysis and/or pleural biopsy confirmed the malignant origin of the effusion in 99 patients (76 carcinomas, nine pleural mesotheliomas and 14 non-epithelial malignancies). Effusions were confirmed as benign in 97 patients (33 cardiac failures, 39 infectious diseases – including 12 tuberculosis – and 25 miscellaneous effusions). Both markers were significantly higher in malignant than in benign effusions. All the patients with non-epithelial malignancies presented CYFRA and CEA values lower than the 95% diagnostic specificity thresholds (100 and 6 ng ml$^{-1}$ respectively). The diagnostic sensitivity in the group of carcinomas and mesotheliomas was similar for CYFRA (58.8%) and CEA (64.7%). However, CEA had a significantly higher sensitivity in carcinomas (72.4% vs 55.3%), while CYFRA had a clearly higher sensitivity in mesotheliomas (89.9% vs 0%). Interestingly, 12 out of the 16 malignant effusions with a negative cytology were CEA and/or CYFRA positive. Regarding their high diagnostic sensitivity and their complementarity, CEA and CYFRA appear to be very useful for the diagnosis of malignant pleural effusions when cytology is negative.

**Keywords:** pleural effusion; carcinoembryonic antigen; CYFRA 21-1; tumour marker; mesothelioma

The aetiological diagnosis of pleural effusions remains an important clinical problem. Cytological analysis detects neoplastic cells in about 60% of the effusions occurring in the course of malignancies (Johnston, 1985; Serre et al, 1990). When the cells present in an effusion cannot be identified as malignant from morphological and cytochemical criteria only, immunocytochemical labelling with monoclonal antibodies specific for various tumour-associated antigens often allows their identification and thus slightly increases the diagnostic sensitivity of cytological analysis (Daste et al, 1991). In the remaining cases, when cytology does not permit a conclusion, blind pleural biopsy or biopsy under thoracoscopy must be used (Loddenkemper and Boutin, 1993; Harris et al, 1995). Determination of various tumour markers in pleural fluid, particularly CEA, has been proposed as a less invasive procedure to improve the biological diagnosis of malignant effusions (Rapellino et al, 1990; Villena et al, 1996).

CYFRA 21-1, a new tumour marker assay measuring soluble fragments of cytokeratin 19, has recently been described (Bodenmüller et al, 1992). Cytokeratin 19 is a major component of the cytoskeleton intermediate filaments of simple epithelium cells and is overexpressed in various carcinomas (Moll et al, 1982). A series of concordant studies showed that CYFRA is very suitable for the diagnosis and the follow-up of non-small-cell lung carcinomas, particularly squamous cell carcinomas (Pujol et al, 1993; Stieber et al, 1993; Van der Gaast et al, 1994). High serum values of CYFRA have also been described in other squamous cell carcinomas and in various adenocarcinomas. Thus, the serum performances of CYFRA in lung cancer and its broad spectrum led us to evaluate its usefulness in the diagnosis of pleural malignant effusions in comparison with CEA and cytology.

*Received 1 April 1997*
*Revised 27 June 1997*
*Accepted 9 July 1997*

*Correspondence to:* G Serre, Laboratoire de Biologie Cellulaire et Cytologie, CHU Purpan, Place du Dr Baylac, 31059 Toulouse Cedex, France

### MATERIALS AND METHODS

### Patients

We retrospectively studied 196 pleural effusions collected from patients of the Department of Pneumology and Internal Medicine of Purpan Hospital in Toulouse, France (Table 1). A definite diagnosis was available for all the patients. Malignant pleural involvement was ascertained in 99 patients (54.5% men, aged from 25 to 85 years, median 65 years) by the presence of malignant cells in pleural fluid and/or in pleural biopsy (blind needle biopsy or biopsy under thoracoscopy). In a control group of 97 patients (57.7% men, aged from 11 to 89 years, median 73 years), benign disease was diagnosed and confirmed by follow-up and/or efficiency of a specific treatment. These two groups were representative of the main causes of pleurisies (Serre et al, 1990; Villena et al, 1996).

### Cytological analysis and tumour marker assay

Pleural fluid was obtained by thoracocentesis, collected in sterile tubes without anticoagulant and rapidly brought to the laboratory. After performing a cell count of the sample of pleural fluid with a haemocytometer, optimal dilution was carried out to obtain 300 nucleated cells per µl and several samples of 0.7 ml were cytocentrifuged at 700 r.p.m. for 17 min in a Cytospin 2 (Shandon,

*The first two authors contributed equally to the work.

Cheshire, UK). Air-dried slides were stained with the May–Grünwald–Giemsa method, and absolute ethanol-fixed slides were stained with the Papanicolaou method, for morphological examinations. Periodic acid–Schiff and Alcian Blue (pH 2.5) cytochemical reactions and immunocytochemical analysis were also performed on the slides to aid the characterization of suspect and malignant cells (Daste et al, 1991).

Part of the sample was centrifuged and the supernatant was aliquoted and stored at –80°C until tumour marker assay. CEA and CYFRA were assayed in duplicate using two commercial enzyme immunoassays (MEIA CEA, Abbott, France, and Enzymum Test CYFRA 21-1, Boehringer Mannheim, France). Dilutions were carried out, if necessary, with the appropriate diluent as recommended by the manufacturer.

### Data analysis

For both tumour markers, pleural thresholds were defined for a diagnostic specificity of 95%, i.e. 5% of false positives in the group of benign effusions (Stieber et al, 1993; Van der Gaast et al, 1994). The sensitivities, at this level of specificity, were calculated in various groups of malignant effusions and compared using the $\chi^2$ test (or Fisher's exact test according to the size of the groups). Receiver-operating characteristic curves were also constructed for CEA and CYFRA by calculating sensitivities and specificities for several cut-off points, and the areas under the curves were compared (Hanley and McNeil, 1983). Differences between groups were tested using the Mann–Whitney *U*-test. Correlations were sought by calculating Spearman's rank correlation coefficient. Differences were considered significant for $P \leq 0.05$.

**Table 1** Sample of patients

| Diagnosis | Number |
|---|---|
| Carcinoma ($n$ = 76) | |
| Lung adenocarcinoma | 18 |
| Small-cell lung carcinoma | 6 |
| Squamous cell lung carcinoma | 2 |
| Breast adenocarcinoma | 12 |
| Digestive adenocarcinoma | 6 |
| Adenocarcinoma of unknown primary site | 27 |
| Other carcinomas[a] | 5 |
| Mesothelioma ($n$ = 9) | |
| Non-epithelial malignancies ($n$ = 14) | |
| Lymphoma/leukaemia | 12 |
| Sarcoma | 2 |
| Benign ($n$ = 97) | |
| Cardiac failure | 33 |
| Parapneumonic | 20 |
| Tuberculosis | 12 |
| Systemic diseases[b] | 10 |
| Empyema | 7 |
| Liver cirrhosis | 4 |
| Other[c] | 11 |

[a]Including ovary (two), kidney (one) adenocarcinoma and head and neck squamous cell carcinoma (two). [b]Including rheumatoid arthritis (four), systemic lupus erythematosus (three), Gougerot-Sjögren's syndrome (one), scleroderma (one) and Sharp's syndrome (one). [c]Including post-traumatic (four), pulmonary embolism (three), benign asbestosis (two), sarcoidosis (one) and nephrotic syndrome (one).

### RESULTS

### Comparison of CEA and CYFRA in benign and malignant effusions

The distribution of CEA and CYFRA values in benign and malignant effusions is represented in Figure 1. A significant but very weak correlation was found between the two tumour markers both in the benign and in the malignant effusions ($r$ = 0.29, $P$ < 0.04 and $r$ = 0.22, $P$ < 0.03 respectively). For CEA, the median was 1.0 ng ml$^{-1}$ (range 0.2–36.8) in the benign group, while it was 8.5 ng ml$^{-1}$ (range 0.5–6760) in the malignant effusions. For CYFRA, the median was 19.5 ng ml$^{-1}$ (range 0.5–332) in the benign group and 101 ng ml$^{-1}$ (range 2–26 600) in the malignant effusions. The distribution of the values of each marker appeared significantly higher in the malignant than in the benign effusions ($P < 10^{-5}$).

### Diagnostic specificity

The thresholds, corresponding to a diagnostic specificity of 95%, were 6.0 ng ml$^{-1}$ for CEA and 100 ng ml$^{-1}$ for CYFRA. None of the false positives for one marker was a false positive for the other. For CEA, these false positives were encountered in non-tuberculous infectious pleurisies and for CYFRA in two patients with cardiac failure, one with tuberculosis and two with empyema. Interestingly, the two highest values among the false positives were observed in a context of empyema (9.9 and 36.8 ng ml$^{-1}$ for CEA, 208 and 332 ng ml$^{-1}$ for CYFRA).

### Diagnostic sensitivity

The diagnostic sensitivities of CEA, CYFRA and cytology on the whole population of malignant effusions or in various groups of patients classified by the histological origin of their cancer are summarized in Table 2.

**Figure 1** Distribution of pleural fluid concentrations of CEA and CYFRA in the groups of benign effusions (○), non-epithelial malignancies (◆), carcinomas (■) and mesotheliomas (●). The dotted lines correspond to the respective 95% specificity thresholds

**Table 2** Diagnostic sensitivity (%) of cytology, CEA and CYFRA according to the histological type of cancer (with a specificity of 100% for cytology and of 95% for tumour markers)

| Neoplasm | Number | Cytology | CEA | CYFRA |
|---|---|---|---|---|
| All | 99 | 83.8 | 55.6 | 50.5 |
| Lymphoma or sarcoma | 14 | 78.6 | 0 | 0 |
| Carcinoma or mesothelioma | 85 | 84.7 | 64.7 | 58.8 |
| Carcinoma | 76 | 89.5 | 72.4[a] | 55.3[a] |
| Adenocarcinoma | 66 | 87.9 | 71.2 | 59.1 |
| Small-cell lung carcinoma | 6 | 100 | 66.7 | 0 |
| Squamous cell carcinoma | 4 | 100 | 100 | 75 |
| Mesothelioma | 9 | 44.4 | 0* | 89.9[a] |

[a]Significant difference between CEA and CYFRA sensitivities.

**Figure 2** Receiver operating characteristic curves for CEA and CYFRA considering the group of benign effusions for specificity and the group of carcinomas and mesotheliomas for sensitivity

Among the 99 patients with a malignant effusion, 83 had a positive cytology (sensitivity of 83.8%). For the remaining 16 patients, malignancy was confirmed histologically. The sensitivities of CEA and CYFRA in malignant effusions were not significantly different (55.6% and 50.5% respectively). All the patients with lymphomas or sarcomas had low values for both markers, clearly under the 95% specificity thresholds. Consequently, when only the carcinoma and mesothelioma group was considered, the sensitivities of CEA and CYFRA were found to be higher but always non-significantly different (64.7% and 58.8% respectively). We also used receiver-operating characteristic curves to compare the performances of CEA and CYFRA in this group (Figure 2). The similar appearance of the two curves was confirmed by the comparison of the areas under the curves, which did not reveal any significant differences between CEA and CYFRA. However, in the group of carcinomas, CEA was more effective than CYFRA (sensitivity of 72.4% vs 55.3%, $P = 0.04$). Inversely, in the group of mesotheliomas, CYFRA was clearly more discriminant (sensitivity of 89.9% vs 0%, $P = 0.0004$). In mesotheliomas, CYFRA ranged from 17.2 to 10 120 ng $ml^{-1}$ with a median of 201 ng $ml^{-1}$, while, in carcinomas, CYFRA ranged from 6.1 to 26 600 ng $ml^{-1}$ with a lower median of 124 ng $ml^{-1}$. Despite the small size of the groups of patients with small-cell lung carcinomas or squamous cell carcinomas, it is noteworthy that both markers appeared to be frequently elevated in the group of squamous cell carcinomas, while only CEA was high in small-cell lung carcinomas (Table 2). Considering the groups of patients with malignant pleural effusions associated with lung and breast carcinomas, no significant differences were found between CEA and CYFRA sensitivities.

The combination of CEA and CYFRA clearly improved the diagnosis sensitivity as 73.3% of malignant effusions and 85.5% of carcinoma effusions were positive with at least one marker. In effusions with a positive cytology, sensitivity of CEA was 60.2%, while 49.4% of the patients had CYFRA values over the threshold. More interesting was the observation of 12 elevated CEA and/or CYFRA values in the 16 cytologically negative malignant effusions (Table 3). The two markers brought additional information but CYFRA appeared to be more contributive than CEA as a result of its performance with mesotheliomas. Finally, the association of cytology, CEA and CYFRA allowed the best results to be obtained as only four malignant effusions out of 99 (two lymphomas, one sarcoma and one carcinoma) remained negative.

### DISCUSSION

Several authors have suggested using the pleural fluid assay of different tumour markers to improve the cytological diagnosis of malignant pleural effusions. The purpose of this study was to evaluate the interest of the new tumour marker CYFRA 21-1, which is well documented as a serum marker, while only a few studies have been published to date concerning pleural effusions (Satoh et al, 1995; Romero et al, 1996; Toumbis et al 1996). In the present work, we compare the diagnostic performance of CYFRA to those of cytology and CEA, in 196 patients with a perfectly defined diagnosis, consisting of a control group of 97 benign effusions and a group of 99 malignant effusions.

The two markers were weakly correlated, probably because they belong to different families of tumour markers. CEA is a well-known oncofetal protein, while CYFRA corresponds to fragments of cytokeratin 19, an intermediate filament protein mainly expressed in the cytoskeleton of normal simple epithelial (Moll et al, 1982).

Our results regarding CEA (55.6% sensitivity) are concordant with previous data that reported sensitivities of around 50–60% in malignant effusions (Rapellino et al, 1990; Villena et al, 1996). The highest sensitivity was logically observed in carcinomas, while very low values were found in mesotheliomas, in agreement with previous studies (Ebert et al, 1990; Mezger et al, 1990).

**Table 3** Efficiency of CEA and CYFRA in the 16 malignant pleural effusions with a negative cytology

| | | Marker(s) over the pleural threshold[a] | | |
|---|---|---|---|---|
| Neoplasm | Number | CEA | CYFRA 21-1 | CEA and/or CYFRA |
| Lung | 1 | 1 | 1 | 1 |
| Breast | 1 | 0 | 1 | 1 |
| Unknown primary | 6 | 4 | 2 | 5 |
| Mesothelioma | 5 | 0 | 5 | 5 |
| Non-epithelial | 3 | 0 | 0 | 0 |
| Total | 16 | 5 | 9 | 12 |

[a]CEA 6 ng $ml^{-1}$ and CYFRA 100 ng $ml^{-1}$.

© Cancer Research Campaign 1998

Compared with the values usually observed in the serum of patients with benign diseases, the values of CYFRA in benign effusions appeared to be very high. Indeed, our 95% specificity threshold was 100 ng $ml^{-1}$, while, in serum, the widely accepted cut-off in patients with benign diseases is situated at around 3–4 ng $ml^{-1}$ (Pujol et al, 1993; Van der Gaast et al, 1994; Plebani et al, 1995). These large amounts of CYFRA in pleural fluid most probably originate from mesothelial cells in which cytokeratin 19 is strongly expressed (Larocca and Rheinwald, 1984). In the same way, high levels of TPA (tissue polypeptide antigen), a tumour marker corresponding to cytokeratins 8, 18 and 19, have been reported in benign pleural effusions (Parazzi et al, 1987; Tokuyama et al, 1995). High values of CYFRA and a similar threshold (around 90 ng $ml^{-1}$ at 95% specificity) were also described in benign effusions (Toumbis et al, 1996), while Satoh et al (1995) and Romero et al (1996) reported a lower cut-off (21 ng $ml^{-1}$ and 50 ng $ml^{-1}$ respectively) but with a specificity of only 71.4% and 82% respectively. However, although high values were observed in benign effusions, CYFRA was significantly higher in malignant effusions and we noted a reasonable sensitivity (50% at 95% specificity), very close to that of CEA. In accordance with the non-expression of CEA and cytokeratins in lymphoma and sarcoma cells, both markers were clearly under the thresholds in these groups. Consequently, when we only considered the group of carcinomas and mesotheliomas, the sensitivity of CEA and CYFRA appeared to be significantly higher (64.7% and 58.8% respectively), reaching the levels of sensitivity previously described for various tumour markers, such as CEA, CA 72-4 or CA 15-3 (Ferroni et al, 1990; Rapellino et al, 1990; Villena et al, 1996). Satoh et al (1995) and Toumbis et al (1996) described similar results for CYFRA, but Satoh reported a higher sensitivity for CYFRA than for CEA. On the other hand, Romero et al (1996) observed a sensitivity of only 38% for CYFRA, in a small series of 41 malignant effusions. Thus, our results agree with those of Satoh et al (1995) and Toumbis et al (1996), and we confirm that CYFRA is one of the most efficient tumour markers available for the diagnosis of malignant effusions.

Here, we also studied the usefulness of CEA and CYFRA in comparison with cytological analysis. CEA and CYFRA were frequently high in patients exhibiting a malignant cytology but the most striking fact was the demonstration of 12 elevated CEA and/or CYFRA values out of the 16 cytologically negative malignant effusions. In fact, the combination of cytology and/or CEA and/or CYFRA allowed the diagnosis of 95 out of 99 malignant effusions. Moreover, among the four false negatives, we found three non-epithelial malignancies. Thus, with the exception of empyemas, which are infrequently associated with malignant effusions but in which tumour markers may be falsely increased (and so must be avoided), these easily performed and non-invasive assays represent a very sensitive association that may alert and help cytologists when cytology remains suspect or negative. They also constitute a good argument for physicians to rapidly perform new thoracocentesis and/or thoracoscopy.

The good diagnostic efficiency of the association CEA/CYFRA in the group of carcinomas and mesotheliomas is directly dependent on their complementarity. Indeed, CEA is more effective in carcinomas and notably in small-cell lung carcinomas, while CYFRA is also sensitive in carcinomas but, above all, very sensitive in mesotheliomas. The high performance of CYFRA in carcinomas was expected because elevated levels of CYFRA had been described in the serum of patients with adenocarcinomas of the lung (Pujol et al, 1993; Stieber et al, 1993; Van der Gaast et al, 1994; Plebani et al, 1995), stomach (Nakata et al, 1996), ovary (Inaba et al, 1996), bladder (Senga et al, 1996) and breast (Molina et al, 1994). Moreover, CYFRA is presented as the most sensitive serum tumour marker in squamous cell lung carcinomas (Pujol et al, 1993; Stieber et al, 1993; Van der Gaast et al, 1994; Plebani et al, 1995) and is of potential interest in squamous cell carcinomas of the uterine cervix (Ferdeghini et al, 1994) or head and neck (Doweck et al, 1995). In pleural effusions, Toumbis et al (1996) and Satoh et al (1995) observed a high sensitivity for CYFRA in squamous cell lung carcinomas. Despite the small size of the group of squamous cell carcinomas, our results lead to the same conclusion.

On the other hand, this study is the first to describe the great sensitivity of pleural CYFRA in mesothelioma diagnosis. This agrees with the expression of cytokeratin 19 in mesothelioma cells (Larocca and Rheinwald, 1984) and with the high TPA values in the pleural fluid of patients with mesothelioma (Parazzi et al, 1987; Tokuyama et al, 1995). Pleural mesotheliomas are malignancies of increasing frequency (Peto et al, 1995), and their cytological and histological diagnosis remains difficult. Although our results indicate that the diagnosis of mesothelioma must be suspected when a low CEA is associated with a high CYFRA, this profile is not specific because some adenocarcinomas have the same profile. In agreement with Villena et al (1996), who recently reported high CA 15-3 levels in pleural fluid of seven out of ten patients with mesothelioma, our preliminary unpublished data seem to confirm the potential interest of this pleural marker in mesotheliomas. Hyaluronic acid is also known to have a good specificity in mesothelioma (Ebert et al, 1990). Hence, low CEA values associated with high values of CYFRA, CA 15-3 and hyaluronic acid would strongly suggest a mesothelioma.

In conclusion, our results confirm the high sensitivity of pleural fluid CYFRA in various carcinomas and underscore its great interest in mesotheliomas. We suggest that the association of CEA and CYFRA is very useful in the diagnosis of malignant effusions, particularly when cytological analysis does not identify malignant cells and when the clinical context is compatible with a malignancy.

### ACKNOWLEDGEMENTS

This work was supported in part by grants from the 'Association pour la Recherche contre le Cancer'. The authors are very grateful to Abbott France and to Boehringer Mannheim, France, for providing diagnostic kits. The authors also thank J Granié, P Gauthier and H Bagat for their excellent technical assistance.

### REFERENCES

Bodenmüller H, Banauch D, Ofenloch-Hähnle B, Jaworek D and Dessauer A (1992) Technical evaluation of a new automated tumour marker assay: the Enzymun-Test CYFRA 21-1. In *Tumor Associated Antigen, Oncogenes, Receptors, Cytokeratins in Tumor Diagnostic and Therapy at the beginning of the Nineties*, Klapdor R. (ed.), pp. 137–138. W Zuckshwerds: Munich

Daste G, Serre G, Mauduyt MA, Vincent C, Caverivière P and Soleilhavoup JP (1991) Immunophenotyping of mesothelial cells and carcinoma cells with monoclonal antibodies to cytokeratins, vimentin, CEA and EMA improves cytodiagnosis of serous effusions. *Cytopathology* **2**: 19–28

Doweck I, Barak M, Greenberg E, Uri N, Kellner J, Lurie M and Gruener N (1995) A new potential tumor marker for squamous cell carcinoma of head and neck. *Arch Otolaryngol Head Neck Surg* **121**: 177–181

Ebert W, Stabrey A, Sibinger M and Shrenk M (1990) Value of pleural fluid hyaluronic acid and carcinoembryonic antigen determinations in differential diagnosis between malignant mesothelioma and pleuritis carcinomatosis. *Tumordiagn Ther* **12**: 1–6

Ferdeghini M, Gaducci A, Prontera C, Castellani C, Annicchiarico C, Gagetti O, Facchini V and Bianchi R (1994) Determination of serum levels of different cytokeratins in patients with uterine malignancies. *Anticancer Res* **14**: 1393–1398

Ferroni P, Szpak C, Greiner JW, Simpson JF, Guadagni F, Johnston WW and Colcher D (1990) CA 72-4 radioimmunoassay in the diagnosis of malignant effusions: comparison of various tumor markers. *Int J Cancer* **46**: 445–451

Hanley JA and McNeil BJ (1983) A method for comparing the areas under receiver operating characteristic curves derived from the same cases. *Radiology* **148**: 839–843

Harris RJ, Kavuru MS, Rice TW and Kirby TJ (1995) The diagnostic and therapeutic utility of thoracoscopy. *Chest* **108**: 828–841

Inaba N, Negishi Y, Fukasawa I, Okajima Y, Ota Y, Tanaka K, Matsui H, Iwasaki H, Sudo H and Tanaka N (1995) Cytokeratin fragment 21-1 in gynecologic malignancy: comparison with cancer antigen 125 and squamous cell carcinoma-related antigen. *Tumor Biol* **16**: 345–352

Johnston WW (1985) The malignant pleural effusion. A review of cytopathologic diagnoses of 584 specimens from 472 consecutive patients. *Cancer* **56**: 905–909

Larocca PJ and Rheinwald JG (1984) Coexpression of simple epithelial keratins and vimentin by human mesothelium and mesothelioma in vivo and in culture. *Cancer Res* **44**: 2991–2999

Loddenkemper R and Boutin C (1993) Thoracoscopy: present diagnostic and therapeutic indications. *Eur Respir J* **6**: 1544–1555

Mezger J, Lamerz R and Permanetter W (1990) Diagnostic significance of carcinoembryonic antigen in the differential diagnosis of malignant mesothelioma. *J Thorac Cardiovasc Surg* **100**: 860–866

Molina R, Agusti C, Filella X, Jo J, Joseph J, Gimenez N and Ballesta AM (1994) Study of a new tumor marker, Cyfra 21-1, in malignant and non malignant diseases. *Tumor Biol* **15**: 318–325

Moll R, Franke WW, Schiller DL, Geiger P and Krepler R (1982) The catalog of human cytokeratins: patterns of expression in normal epithelia, tumors and cultured cells. *Cell* **31**: 11–24

Nakata B, Chung YS, Kato Y, Ogawa M, Ogawa Y, Inui A, Maeda K, Sawada T and Sowa M (1996) Clinical significance of serum CYFRA 21-1 in gastric cancer. *Br J Cancer* **73**: 1529–1532

Parazzi F, Faravelli B, Gallo L, Nosenzo M, Razzetti A, Barone D, Bandelloni R and D'Amore E (1987) Tissue polypeptide antigen (TPA) in pleural effusions. *Tumori* **73**: 33–36

Peto J, Hodgson JT, Matthews FE and Jones JR (1995) Continuing increase in mesothelioma mortality in Britain. *Lancet* **345**: 535–9

Plebani M, Basso D, Navaglia F, De Paoli M, Tommasini A and Cipriani A (1995) Clinical evaluation of seven tumour markers in lung cancer diagnosis: can any combination improve the results? *Br J Cancer* **72**: 170–173

Pujol JL, Grenier J, Daurès JP, Daver A, Pujol H and Michel FB (1993) Serum fragment of cytokeratin subunit 19 measured by CYFRA 21-1 immunoradiometric assay as a marker of lung cancer. *Cancer Res* **53**: 61–66

Rapellino M, Pecchio F, Piantino P, Cellerino A, Baldi S, Scapaticci E, Libertucci D, Coni F, Adamo MR, Arosio C, Ruffini E and Cianci R (1990) Tumor markers determination in extrahematic fluids. *J Nucl Med Allied Sci* **34**: 128–136

Romero S, Fernandez C, Arriero JM, Espassa A, Candela A, Martin C and Sanchez-Paya J (1996) CEA, CA 15-3 and CYFRA 21-1 in serum and pleural fluid of patients with pleural effusions. *Eur Respir J* **9**: 17–23

Satoh H, Sumi M, Yagyu H, Ishikawa H, Suyama T, Naitoh, Saitoh T and Hasegawa S (1995) Clinical evaluation of CYFRA 21-1 in malignant pleural fluids. *Oncology* **52**: 211–214

Senga Y, Kimura G, Hattori T and Yoshida K (1996) Clinical evaluation of soluble cytokeratin 19 fragments (CYFRA 21-1) in serum and urine of patients with bladder cancer. *Urology* **48**: 703–710

Serre G, Daste G, Vincent C, Mauduyt MA and Soleilhavoup JP (1990) Diagnostic approach to the patient with pleural effusion: cytologic analysis of pleural fluid. In *International Trends In General Thoracic Surgery*, Vol. 6, Deslauriers J and Lacquet LK. (ed), pp. 35–45. The CV Mosby Company: St Louis

Stieber P, Hasholzner U, Bodenmüller H, Nagel D, Sunder-Plassmann L, Dienemann H, Meier W and Fateh-Moghadam A (1993) CYFRA 21-1: a new marker in lung cancer. *Cancer* **72**: 707–713

Tokuyama T, Yoneda T, Hamada K, Yoshikawa M, Fu A, Tomoda M, Nakaya N, Tamura M and Kitamura K (1995) Diagnostic value of tissue polypeptide antigen in pleural effusions with malignant mesothelioma. *Nippon Kyobu Shikkan Gakkai Zasshi* **33**: 39–43

Toumbis M, Rasidakis A, Passalidou E, Kalomenidis J, Alchanatis M, Orphanidou D and Jordanoglou J (1996) Evaluation of CYFRA 21-1 in malignant and benign pleural effusions. *Anticancer Res* **16**: 2101–2104

Van der Gaast A, Schoenmakers CHH, Kok TC, Blijenberg BG, Cornillie F and Splinter TAW (1994) Evaluation of a new tumor marker in patients with non-small-cell lung cancer: Cyfra 21-1. *Br J Cancer* **69**: 525–528

Villena V, Lopez-Encuentra A, Echave-Sustaeta J, Martin-Escribano P, Ortuno-De-Solo B and Estenoz-Alfaro J (1996) Diagnostic value of CA 72-4, carcinoembryonic antigen, CA 15-3 and CA 19-9 assay in pleural fluid. *Cancer* **78**: 736–740

© Cancer Research Campaign 1998